# Ketoacidosis and SGLT2 Inhibitors: A Narrative Review

**DOI:** 10.3390/metabo14050264

**Published:** 2024-05-06

**Authors:** Carmela Morace, Giuseppe Lorello, Federica Bellone, Cristina Quartarone, Domenica Ruggeri, Annalisa Giandalia, Giuseppe Mandraffino, Letteria Minutoli, Giovanni Squadrito, Giuseppina T. Russo, Herbert Ryan Marini

**Affiliations:** 1 Department of Clinical and Experimental Medicine, University of Messina, 98125 Messina, Italy; carmela.morace@unime.it (C.M.); federica.bellone@unime.it (F.B.); gmandraffino@unime.it (G.M.); lminutoli@unime.it (L.M.); gsquadrito@unime.it (G.S.); giuseppina.russo@unime.it (G.T.R.); 2 Lipid Clinic and Cardiometabolic Disease Center, University Hospital of Messina, 98124 Messina, Italy; 3 Internal Medicine and Diabetology Unit, University Hospital of Messina, 98124 Messina, Italy; giuseppelorello@alice.it (G.L.); cristina.quartarone@polime.it (C.Q.); domenica.ruggeri@polime.it (D.R.); annalisa.giandalia@unime.it (A.G.); 4 Department of Human Pathology of Adulthood and Childhood “G. Barresi”, University of Messina, 98125 Messina, Italy

**Keywords:** diabetic ketoacidosis, SGLT2-i, adverse drug reaction

## Abstract

An acute metabolic complication of diabetes mellitus, especially type 1, is diabetic ketoacidosis (DKA), which is due to an increase in blood ketone concentrations. Sodium/glucose co-transporter-2 inhibitor (SGLT2-i) drugs have been associated with the occurrence of a particular type of DKA defined as euglycemic (euDKA), characterized by glycemic levels below 300 mg/dL. A fair number of euDKA cases in SGLT2-i-treated patients have been described, especially in the last few years when there has been a significant increased use of these drugs. This form of euDKA is particularly insidious because of its latent onset, associated with unspecific symptomatology, until it evolves (progressing) to severe systemic forms. In addition, its atypical presentation can delay diagnosis and treatment. However, the risk of euDKA associated with SGLT2-i drugs remains relatively low, but it is essential to promptly diagnose and manage it to prevent its serious life-threatening complications. In this narrative review, we intended to gather current research evidence on SGLT2i-associated euDKA from randomized controlled trials and real-world evidence studies, its diagnostic criteria and precipitating factors.

## 1. Introduction

Diabetes mellitus (DM) is a group of metabolic diseases characterized by hyperglycemia resulting from defects in insulin secretion, insulin action, or both [1]. It is one of the most important growing diseases worldwide and a prominent public health burden. Based on the International Diabetes Federation (IDF) Atlas data, the global diabetes prevalence in adult populations in 2021 was estimated to be almost 10.5% (536.6 million people), rising to 12.2% (783.2 million) by 2045 [2]. DM is a complex disease and its management requires multifactorial behavioral and pharmacological treatments to maintain quality of life and prevent or delay complications [3]. In fact, diabetes complications are the leading cause of morbidity and mortality in individuals with diabetes. These are commonly divided into acute and chronic complications. The chronic diabetes complications are both macro- (cardiovascular disease) and microvascular diseases (diabetic kidney disease, diabetic retinopathy and neuropathy) [4]. The acute complications include diabetic ketoacidosis (DKA) and non-ketotic hyperosmolar state (NKHS); while the first is primarily seen in individuals with type 1 DM (T1DM) and the latter is prevalent in people with type 2 DM (T2DM), both disorders can be found in both diabetes types. Unlike chronic complications, which develop gradually, DKA and NKHS can quickly evolve into severe forms if not readily diagnosed and treated [5].

### 1.1. Diabetic Ketoacidosis

DKA is one of the most serious acute metabolic complications of DM. It occurs as a consequence of severe absolute or relative insulin deficiency, which results in a low glucose uptake in insulin-dependent tissues (muscle, liver and fat) [6]. This alteration of glucose metabolism triggers a counter-regulatory insulin response, lipolysis stimulation and an augmented efflux of free fatty acids (FFAs). The hepatic oxidation of FFA induces the synthesis and accumulation of ketones, owing to the citric acid cycle failure [7]. DKA usually develops in association with precipitating factors. The most common are infection, the discontinuation of or inadequate insulin therapy, pancreatitis, myocardial infarction and cerebrovascular accidents [8]. DKA is frequent in patients with type T1DM, but it can also develop in patients with T2DM [9].

To date, there have been conflicting clinical definitions for DKA diagnosis, and so far, no definitive criteria have been drawn up. No consensus is present on parameters such as ketonemia/ketonuria, HCO_3_^−^, pH and glucose values [10,11].

The symptoms of poorly controlled diabetes may be present for several days or weeks, but the metabolic alterations typical of DKA usually evolve in a short time (typically <24 h). Sometimes, the entire symptomatic presentation may evolve or develop more acutely, with no previous clue or symptom. The clinical appearance includes polyuria, polydipsia, weight loss, vomiting, dehydration and weakness. Physical findings may include poor skin turgor, deep hyperventilation (Kussmaul respirations in severe cases), acetone on breath tachycardia and hypotension. Mental status can vary from full alertness to profound lethargy or coma; focal neurologic signs (hemianopia and hemiparesis) and seizures (focal or generalized) may also be present. Although infection is a common precipitating factor, patients can be normothermic or even hypothermic because of peripheral vasodilation (severe hypothermia is a poor prognostic sign). Nausea, vomiting and diffuse abdominal pain are frequent; the latter can be a symptom of ketosis or indicate a precipitating cause of DKA [8,12].

The diagnostic criteria for DKA issued by different scientific societies are different.

The diagnostic criteria for DKA indicated by the American Diabetes Association (ADA) include the following specific clinical parameters:

- Blood glucose levels >250 mg/dL;
- Arterial blood gases pH <7.3;
- Anion GAP >10 mEq/L;
- HCO_3_^−^ <18 mEq/L;
- Ketones in blood and/or urine.

Depending on the levels of these parameters, mild, moderate and severe DKA are classified [13]. Instead, according to the Joint British Diabetes Societies (JBDS) guidelines, a history of diabetes, independently to glucose levels (even if glucose levels >200 mg/dL are indicated), is sufficient criterion for a diagnosis of DKA. 

With hydroxybutyrate testing at the bedside, the measurement of serum ketones with a level >3 mmol/L has been suggested as part of the diagnostic criteria for ketoacidosis instead of using urine ketones. Furthermore, the UK guidelines recommend testing venous blood gas rather than arterial blood gas, with a pH <7.3 for the diagnosis of acidosis [14,15].

According to the American Association of Clinical Endocrinologists (AACE) and the American College of Endocrinology (ACE), diagnostic criteria for DKA are as follows:

- Arterial pH <7.3;
- β-hydroxybutyrate ≥40 mg/dL (3.8 mmol/L) in adults;
- Ketone-positive urine (nitroprusside reaction method);
- Anion gap <10 mEq/L;
- Drowsy, stupor or coma in moderate to severe DKA [16].

In Italy, a consensus statement on DKA has been recently issued by the Scientific Societies AMD (Associazione Medici Diabetologi), SID (Società Italiana di Diabetologia) and SIEDP (Società Italiana di Endocrinologia e Diabetologia Pediatrica). 

According to the three above mentioned scientific societies, the diagnosis of DKA can be made on the basis of the following biochemical criteria:

- Hyperglycemia: blood glucose levels >11 mmol/L (200 mg/dL);
- Venous pH <7.3 or serum bicarbonate <15 mmol/L;
- Ketonemia and/or ketonuria.

Blood β-hydroxybutyrate (B-OHB) concentration ≥3 mmol/L is indicative of DKA.

Urine ketones are typically moderately or largely positive (≥2+).

DKA severity is classified on the basis of acidosis levels:

- Mild: venous pH < 7.3 or serum bicarbonate <15 mmol/L;
- Moderate: pH < 7.2, serum bicarbonate <10 mmol/L;
- Severe: pH < 7.1, serum bicarbonate <5 mmol/L.

In Table 1, the DKA diagnostic criteria of the JBDS guidelines, ADA guidelines and AACE/ACE guidelines and the AMD, SID and SIEDP consensus statement are compared.

Although DKA is usually associated with marked hyperglycemia, it is possible to observe DKA even when small increases in or normal blood glucose levels are present. This condition was firstly described by Munro et al. (1973) and named euglycemic DKA (euDKA); it differs from classic DKA and is characterized by glycemic levels below 300 mg/L. Indeed, these authors referred a series of 211 events of diabetic metabolic decompensation, 37 of which had severe ketoacidosis indicated by blood glucose levels of <300 mg/dl and plasma HCO_3_^−^ <10 mEq/l [17]. 

The last Joint British Diabetes Societies (JBDS) guideline defined euDKA as the presence of the following:

- Diabetic people with normal or not particularly raised glucose levels;
- The development of raised anion gap metabolic acidosis;
- Ketonemia (>3.0 mmol/L) or significant ketonuria (2+ or more on standard urine sticks) [15].

Table 2 compares the currently used criteria for the diagnosis of euDKA.

To date, the etiopathogenetic mechanism of euDKA is unknown, but the clinical manifestations are similar to DKA, except for the blood glucose levels. As the classic form, euDKA usually develops in association with precipitating factors: fever, infection, concurrent pathologies, the post-operative stage, reduction in caloric and/or fluid intake, alcohol abuse and low-carbohydrate diet. Moreover, a fair number of scientific evidence show a correlation between euDKA and the use of sodium/glucose co-transporter-2 inhibitor (SGLT2-i) drugs [18].

### 1.2. Sodium/Glucose Co-Transporter-2 Inhibitors

The sodium/glucose co-transporters (SGLTs) are involved in the active transport of glucose into renal tubules against a concentration gradient, with the simultaneous co-transport of Na^+^ down concentration gradients [19]. There are two different types of SGLT:

- SGLT type 1 (SGLT1) is a high-affinity low-capacity transporter, encoded by the *SCL5A1* gene. It is located in the S2 and S3 segments of the renal proximal tubule. The percentage of renal glucose absorption by SGLT1 is almost 10%; the ratio of glucose and sodium co-transportation is 1:2. Its principal extrarenal locations are the gastrointestinal tract; in fact, the clinical syndrome resulting from *SCL5A1* gene mutation has diarrhea as the dominant symptom. Heart and red blood cells are other extrarenal sites.
- SGLT type 2 (SGLT2) is a low-affinity high-capacity transporter, encoded by the *SCL5A2* gene. It is present mainly in the S1 segment of the proximal tubule, near Bowman’s capsule. It reabsorbs almost 90% of the filtered glucose; the ratio of glucose and sodium co-transportation is 1:1. Its extrarenal locations are the brain and liver [20,21].

**Figure 1:**
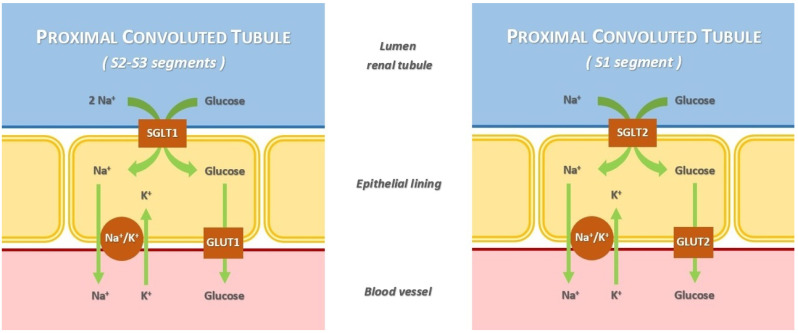
Mechanism of glucose and Na^+^ renal reabsorption mediated by sodium/glucose co-transporters.

The action of SGLT1 and SGLT2 is complementary; their coupling with the specific glucose transporters (GLUT1 and GLUT2) and Na^+^/K^+^-ATPase forms a single mechanism able to recover glucose from the renal filtrate. When the kidney reabsorption capacity is exceeded, glycosuria starts to appear (Figure 1) [22].

SGLTs can reabsorb up to 97% of glucose reabsorption and about 5% of total renal Na^+^; the latter can increase to 14% in diabetic patients. In healthy subjects, maximum reabsorption capacity is obtained when the blood glucose concentration is approximately 200 mg/dL; the SGLTs reabsorb up to a maximum of about 375 mg/min (2.08 mmoL/min) [20]. The SGLT2 inhibitor (SGLT2-i) drugs, blocking this mechanism, induce increased glucose excretion and a reduction in blood glucose and glycated hemoglobin levels [21]. For these reasons, originally SGLT2-i drugs were classified as “antidiabetic drugs”. Moreover, they can be considered diuretics with a “hybrid” mechanism: an osmotic diuresis dependent on glycosuria and a natriuresis [20,23]. Furthermore, in T2DM patients, the altered blood sugar levels increase the quantity of the glucose ultrafiltrated through the glomeruli and its concentration in the renal tubule. It was reported that this condition brings a greater SGLTs expression, resulting in a surge of the glucose reabsorption rate [24]. This attenuated glycosuria leads to increased glucose blood values and a reduced sodium concentration in renal tubules, which causes the activation of the juxtaglomerular apparatus and renin–angiotensin–aldosterone system (RAAS) [22,25]. Therefore, by blocking SGLT2, these drugs reduce either blood glucose levels or the RAAS activation as a consequence of their natriuretic effect. However, the mechanism of action of these drugs has not yet been fully understood [20,26]. 

Indeed, large randomized controlled trials showed the important and consistent effects of SGLT2-i use in patients with T2DM on reductions in death and major adverse cardiac events (MACE) in people with atherosclerotic cardiovascular disease (ASCVD) or high/very high cardiovascular risk. In parallel to cardioprotection, SGLT2-i drugs have been demonstrated to improve renal outcomes and to prevent the progression of kidney disease. Furthermore, treatment with an SGLT2-i is associated with significant reductions in weight, systolic and diastolic pressure and uricemia [27,28,29,30,31]. In the last few years, the efficacy of SGLT2-i drugs in reducing all-cause and cardiovascular death and hospitalizations for heart failure in patients affected by heart failure with reduced and preserved ejection fraction (HFrEF/HFpEF), regardless of the T2DM presence, has been highlighted [32]. Recent studies provided evidence that SGLT2-i use could be effective also on Non- Alcoholic Fatty Liver Disease (NAFLD)/Non-Alcoholic Steatohepatitis (NASH) in patients with T2DM by reducing liver fat accumulation [33,34]. Accordingly, SGLT2-i use in T2DM patients is increasing overtime [35].

Despite these important effects, SGLT2-i use is not free from side effects. The most common adverse events are mild–moderate genital infections, especially in women and in patients with previous events of genital infection or predisposing conditions (e.g., benign prostatic hypertrophy); genital infections usually respond well to the conventional therapy. Moreover, due to their diuretic effect, SGLT2-i use may cause volume depletion, mainly in elderly patients [27,28,29,30,31]. In CANVAS trial, there has been a slight increase in the incidence of minor foot amputations [28], although, to date, this evidence appears to have been disproved [31,32,33,34,35,36]. 

Furthermore, after the introduction of SGLT2-i use, some rare but serious cases of euDKA were described in patients administered with this class of drugs. Because of this, for the first time in May 2015 (subsequently updated in 2019 and in 2020), the Food and Drug Administration (FDA) published a safety announcement of 73 cases of ketoacidosis in T1DM or T2DM patients treated with SGLT2-i drugs from March 2013 (date of the first drug of the class) to May 2015 [37]. In many of the above-mentioned cases, DKA was recognized later, owing to only mild glucose elevations, lower than that expected for typical DKA [38]. On February 2016, recommendations to minimize the risk of diabetic ketoacidosis in patients treated with SGLT2-i drugs were confirmed by the European Medicines Agency (EMA) [39].

In the AACE and ACE position statement (2016), after the review of more than 80 DKA cases from the literature (including both events involving SGLT2-i drugs and those occurring before their introduction), it was found that in patients treated with SGLT-2 inhibitors, DKA was more frequent in insulin-deficient subjects such as those with T1DM and including latent autoimmune diabetes in adults (LADA), but cases have also occurred in some patients with longstanding T2DM. The lowest recorded blood glucose level was 90 mg/dL, while in 13 cases, the mean value was <180 mg/dL. However, the majority of recorded values were >250 mg/dl, even if blood glucose levels was not reported in many cases [16].

However, euDKA occurs not only in patients treated with SGLT2-i drugs but also in alcohol use disorders, chronic liver disease and pregnancy [17,40].

## 2. Epidemiology

Data on euDKA incidence in SGLT2-i treated patients are obtained from the analysis of large randomized controlled trials (Table 3), although a differentiation between DKA and euDKA is not available in many of these studies. 

Regarding the cardiovascular and renal safety studies on empagliflozin, in the EMPA-REG OUTCOME trial (Empagliflozin Cardiovascular Outcome Event Trial in Type 2 Diabetes Mellitus Patients), DKA events showed a frequency of 3/2342 cases (0.1%) in the group treated with 10 mg of empagliflozin, 1/2345 (<0.1%) case in the empagliflozin 25 mg group and 1/2333 case (<0.1%) in the placebo group [27]. In the EMPEROR-Preserved trial (Empagliflozin Outcome Trial in Patients with Chronic Heart Failure with Preserved Ejection Fraction), ketoacidosis occurred only in patients with diabetes (0.3%), without significant differences between the empagliflozin group and the placebo group [34]. Similarly, in the EMPA-KIDNEY trial (Study of Heart and Kidney Protection with Empagliflozin), the frequency of DKA events was 0.2% in patients administered with 10 mg of empagliflozin and <0.1% in the placebo group [41].

Concerning the dapagliflozin trials, in the DECLARE-TIMI 58 trial (Dapagliflozin Effect on Cardiovascular Events–Thrombolysis in Myocardial Infarction 58), DKA was more frequent in the treatment group than in the placebo group (0.3% vs. 0.1%, *p* value 0.02) [28]. Also, in the DAPA-HF trial (Dapagliflozin and Prevention of Adverse Outcomes in Heart Failure), DKA events were reported in the dapagliflozin group (3/2373, 0.1/100 patients-yr) but not in the placebo group [29]. Similarly, in the DELIVER trial (Dapagliflozin Evaluation to Improve the Lives of Patients with Preserved Ejection Fraction Heart Failure), 6263 patients were analyzed and divided by age categories (age <55, age 55–64, age 65–74 and age >75). In the dapagliflozin group, 2 DKA cases were reported: 1/167 (0.6%) in the age <55 category and 1/1136 (0.1%) in the age 65–74 category; no DKA cases were reported in the placebo group [42]. In the DAPA-CKD trial (Dapagliflozin and Prevention of Adverse Outcomes in Chronic Kidney Disease), 2/2149 cases of ketoacidosis (<0.1%) in patients taking dapagliflozin (10 mg) were reported, and there were no cases in the placebo group (*p* value 0.5) [43].

As for the cardiovascular and renal safety studies on canagliflozin, in the CANVAS (Canagliflozin Cardiovascular Assessment Study) program, DKA cases, even rare ones, were more numerous with canagliflozin (0.6/1000 patients-yr) than with a placebo (0.3/1000 patients-yr) [27]. A higher DKA incidence was also registered in the CREDENCE trial (Canagliflozin and Renal Events in Diabetes with Established Nephropathy Clinical Evaluation): 11/2200 (2.2/1000 patients-years) in the canagliflozin group and 1/2197 (0.2/1000 patients-years) in the placebo arm [30]. When all serious adverse events of DKA were considered, including 17,596 subjects from randomized studies of canagliflozin, the incidence of DKA cases in the trial database was 0.07% (12/17,596). The incidence for each treated group was 0.07% (4/5337), 0.11% (6/5350) and 0.03% (2/6909) with 100 mg canagliflozin, 300 mg canagliflozin and the comparator, respectively. After the diagnosis of a DKA-related event, six patients (three from the 100 mg canagliflozin group, three from the 300 mg canagliflozin group, and none from the comparator) showed autoimmune diabetes (latent autoimmune diabetes of adulthood or T1DM) or GAD65 antibodies. With the exception of the above-indicated 6 patients, the incidences of serious adverse events of DKA and related events by the treatment group of patients with type 2 diabetes were 0.02% (1 of 5334), 0.06% (3 of 5347), and 0.03% (2 of 6909) with the 100mg canagliflozin group, 300 mg canagliflozin group and comparator group, respectively [44].

Finally, a similar frequency of diabetic ketoacidosis was reported for ertugliflozin in the VERTIS-CV trial (the Evaluation of Ertugliflozin Efficacy and Safety Cardiovascular Outcomes Trial): 0.3% in the 5 mg ertugliflozin group, 0.4% in 15 mg group and 0.1% in the placebo group [45].

Overall, the available evidence from randomized controlled clinical trials are consistent with the hypothesis that treatment with SGLT2-i drugs causes a 2- to 4-fold increased risk of ketoacidosis [46]. 

There is little evidence coming from “real world” studies. The rates of SGLT-associated ketoacidosis in the “real world” are unclear, although they may be higher than the data suggested by randomized trials. In fact, a careful selection of study participants, an appropriate education the different definitions of ketoacidosis and the duration of the follow-up of a trial may influence the results [47].

The first analysis assessment of the FDA Adverse Event Reporting System (FAERS) compared DKA reports of acidosis in patients treated with SGLT2-i drugs with patients treated with a DPP4-i (from the date of FDA approval of each drug, 15 May 2015). Having excluded patients with T1DM and focusing on patients with T2DM, SGLT2-i-treated patients showed an almost 7-fold increase in developing DKA. Of the cases reported, 71% provided sufficient information to fit criteria for the diagnosis of euDKA [46,48]. 

A subsequent assessment of FAERS comes from an analysis value of 2028 DKA reports, with a diabetes indication for an SGLT2-i from 2014 to 2016. The proportional reporting ratio (PRR) of DKA in DM patients taking an SGLT2-i vs. those not taking it was 7.9 (confidence interval [95% CI] 7.5–8.4), and it was higher for T1DM (PRR 57.3; 95% CI 49.2–66.6). The most common drugs associated with an SGLT2-i in the DKA reports were metformin and insulin. There was a larger number of women, age and body weight varied widely and the duration of SGLT2-i treatment was also variable; for these reasons, SGLT2-i-associated DKA was not considered limited to any demographic or comorbid subpopulation, and it can occur at any duration of SGLT2-i use. It has been reported that DKA caused death in 37 individuals out of 2397 custom searched unique patients (1.54%) [49]. 

In a recent analysis of the FAERS, a total of 10,195 cases of euDKA (*n* = 1680) and DKA (n = 8515) associated with an SGLT2-i were described. The SGLT2-i was related with a higher frequency of euDKA and DKA compared to other hypoglycemic agents (ROR = 16.69 [95% CI 14.89–18.70], IC = 3.27 [95% CI 2.91–3.66] for euDKA; ROR = 16.44 [95% CI 15.72–17.20], IC = 3.19 [95% CI 3.05–3.34] for DKA). EuDKA (51.9%) was more frequent in male patients, while DKA was in female patients (53.7%). A large number of patients discontinued the treatment (95.5% for euDKA and 93.9% for DKA), and nearly 49.0% (*n* = 3658) of patients had a remission of symptoms after the discontinuation of the SGLT2-i; 2.3% (*n* = 173) had no remission. Hospitalization was necessary for 75.6% (*n* = 6126) of patients after euDKA/DKA [50]. 

Recently, a study designed to evaluate the risk of this complication using the European spontaneous reporting system, the DAPA-KETO study (DAPAgliflozin and KETOacidosis study), demonstrated a higher reporting frequency of ketoacidosis observed with dapagliflozin than with DPP-4 inhibitors or insulin [51]. 

In a retrospective cohort study, using administrative health care data from Canada and a primary care clinical database from the United Kingdom (the Clinical Practice Research Datalink), a cohort of over 350,000 adults with T2DM who received an SGLT2-i or a DPP4-i and more than 500 DKA events were identified. From these data analyses, it was found that SGLT2-i use was associated with an almost 3-fold increase in the risk for DKA compared with DPP4-i use (2.03 versus 0.75 per 1000 person-yr; hazard ratio [HR] 2.85; 95% CI 1.99–4.08). Except for a lower risk in patients with a prior receipt of insulin than in those without, the results were consistent regardless of sex, age and recipient type [52]. 

A cohort study with data from nationwide health and administrative registers in Sweden and Denmark analyzed a cohort of 34,426 patients divided into two equal number groups: the first was composed of new users of SGLT2-i drugs, the second of new users of glucagon-like peptide-1 receptor agonists (GLP1-RAs). The DKA events recorded were 19/17,213 (1.3/1000 events-yr) in the first group and 11/17,213 (0.6/1000 events-yr) in the second. From the analysis of these data, SGLT2-i use, when compared with GLP1-RA, can be correlated with a twofold elevated risk of DKA [53]. A retrospective cohort analysis of a large commercially insured patients’ database in the USA, the Truven MarketScan Commercial Claims and Encounters (CCAE), evaluated 50,220 patients with a new prescription for an SGLT2-i and 90,132 who had received a new prescription for a DPP4-i. After 108 days from the initiation of therapy, SGLT2-i administration induced a 2-fold increase in the risk of DKA compared with patients treated with a DPP4-i (4.9 vs. 2.3 events per 1000 person-years; HR 2.1; 95% CI 1.5–2.9). Some differences were present between the two groups; in fact, patients treated with an SGLT2-i were younger and had fewer coexisting illnesses than those treated with a DPP4-i. However, after propensity score matching to adjust for the two group differences, the HR was superimposable (HR 2.2; 95% CI 1.4–3.6) [54].

A recent retrospective, multicenter, controlled cohort study analyzed 162 cases of DKA (37 SGLT2-i users and 125 non-users) with diabetes. SGLT2-i users were more likely to develop DKA as an inpatient compared with non-SGLT2-i users; SGLT2-i use was associated with a small but significant increased risk of DKA. In SGLT2-i users, fifteen (41%) had peak blood glucose levels <250 mg/dL (14 mmol/L), while there was only one (0.8%) among non-users (*p* < 0.001) [55]. 

By contrast, an observational, retrospective new-prescription cohort study based on the above mentioned Truven CCAE database gave different results. After applying the inclusion criteria, 31,401 new users of an SGLT2-i and 108,966 new users of non-SGLT2-i antihyperglycemic agents (AHAs) were included. It was demonstrated that no statistically significant difference was present between the two groups in DKA incidence [56]. 

Furthermore, based on an analysis of the Danish National Patient Register, SGLT2-i therapy is not related to a significant increase in DKA risk. This study estimated the nationwide incidence of DKA in TD2M, with 20 years of follow-up (between 1 January 1995 and 31 December 2014), reporting an higher occurrence in patients on insulin monotherapy and few adverse events among those prescribed an SGLT2-i and concomitantly prescribed other glucose-lowering drugs and insulin [57]. 

In a nationwide cohort study, data were obtained from the Korean Health Insurance Review and Assessment Service (2013–2017), and 56,325 patients who were started on an SGLT2-i were included and were compared with same number of patients who were started on a DPP4-i during a 3-year follow-up period. The authors concluded that SGLT2-i treatment did not increase the risk of hospitalization for DKA when compared to DPP-4-i (HR 0.956; 95% CI 0.5811.572; *p* value 0.996). Furthermore, the incidence of DKA hospitalization was higher during the first 30 days after SGLT2-i treatment initiation (2.501 events per 1000 person-yr cases), than during the next three years (0.614 events per 1000 person-yr). Moreover, SGLT2-i use increased the risk for DKA in patients with DM microvascular complications (HR 2.044; 95% CI 0.900–4.640; *p* value 0.088) and in those prescribed diuretics (HR 3.648; 95% CI 0.720–18.480; *p* value 0.118), although this was not statistically significant [58]. 

In a study aimed to evaluate the safety and tolerability of empagliflozin in patients with T2DM, data from patients treated with two different doses of the drug [10 mg (4221) and 25 mg (4196)] and with a placebo (4203) in 15 randomized phase I-III trials were assessed. From this analysis, it was found that the number of patients positive for urine ketone levels was higher in the empagliflozin groups than in the placebo group, but the diabetic ketoacidosis adverse event was similar between groups. All patients recovered, with the exception of one participant in the empagliflozin 10 mg group whose outcome was unknown. In the investigators’ opinion, no diabetic ketoacidosis adverse events were referred to the drug [59].

Therefore, the “real world” incidence of both euDKA and DKA is still uncertain, due to the different definition, design and heterogeneity of the patient population studied. Finally, some data derive from meta-analyses. A recent meta-analysis of 130 RCTs screening SGLT2-i safety in elderly T2DM subjects (including 19,986 patients) did not show any difference in the euDKA risk between the SGLT2-i and placebo groups [60]. Similarly, in a meta-analysis involving a total of 10 eligible RCTs including 13,134 patients, no difference in the ketoacidosis risk were demonstrated with SGLT2-i or DPP4-i treatments [61].

Taken together, all these findings suggest a small but greater risk of ketoacidosis than with placebos or other drugs, albeit with conflicting results. Notably, the absolute incidence varies according to different designs and populations of the available studies (from 0.3 to 4.9 events per 1000 patient-years). 

As for the outcomes, a retrospective study evaluated 71 T2DM patients hospitalized with DKA including 16 on SGLT2-i treatment upon admission. During hospitalization, the rates of acute kidney injury, concomitant infections and inpatient mortality among SGLT2-i users were comparable to non-users, but the long-term mortality from any cause was lower among the SGLT2-i users [62]. Although ketoacidosis is considered a complication of people with diabetes, recently, two euDKA cases were reported in patients without T2DM administrated by an SGLT2-i for heart failure with reduced ejection fraction, probably triggered by reduced oral nutrition intake [63]. This evidence opens up new scenarios in the investigation of the possible causes of onset and incidence of euDKA in patients without DM.

## 3. Etiopathogenesis

Ketone bodies (acetone, acetoacetic acid and β-hydroxybutyric acid) are formed in the liver under a carbohydrates shortage from the oxidation of fatty acids. Ketones can be used as a substrate to produce energy in cardiac and skeletal muscle, the intestine, the kidney and the brain when enough glucose is not readily available. They are excreted through breathing as acetone and in urine. When ketone bodies’ production exceeds clearance, they accumulate in blood, leading to ketosis or ketoacidosis if the buffer systems are unable to correct it [64].

DKA results from a combination of absolute or relative insulin action deficiency and glucagon level elevations, which induce a shift to fat metabolism. Reduced insulin action coupled with increased glucagon promote hepatic ketogenesis by free fatty acids (FFAs) β-oxidation and reduced ketone use in other tissues [65].

**Figure 2:**
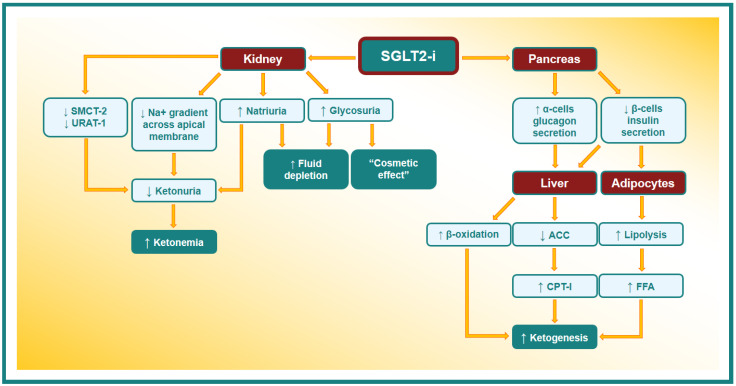
Possible etiopathogenetic mechanism of EuDKA in patients taking SGLT2-i. SMCT-2: sodium-coupled monocarboxylate transporter-2. URAT-1: urate anion transporter 1. ACC: acetyl-CoA carboxylase. CPT-I: carnitine palmitoyltransferase-I. FFA: free fatty acid.

The etiopathogenetic mechanism of euDKA induced by an SGLT2-i is not yet well known, even if different mechanisms have been assumed. The main euDKA etiopathogenetic mechanisms are shown in Figure 2.

An SGLT2-i lowers blood glucose levels by increasing its urinary excretion; this reduces pancreatic β-cells’ insulin secretion. The fall in circulating insulin levels leads to a reduction in the insulin antilipolytic action and consequent stimulation of the free fatty acids (FFAs) production. FFA released from adipocytes are brought to the liver and oriented toward the ketogenic pathway and converted to ketone bodies by β-oxidation [66]. Moreover, insulin stimulates acetyl-CoA carboxylase (ACC) activity, with the production of malonyl-CoA, a strong inhibitor of carnitine palmitoyltransferase-I (CPT-I). CPT-I triggers the FFA transport into mitochondria; in this way, the decrease in the circulating level of insulin promotes the production of ketone bodies through the activation of CPT-I and the β-oxidation rate [67]. 

Furthermore, SGLT2-i administration triggers glucagon secretion through the release of potassium, with the subsequent entry of calcium into pancreatic α-cells [68]. As glucagon inhibits acetyl-CoA carboxylase and increases CPT-I liver activity, the overproduction of ketone bodies can be considered the consequence of increased glucagon secretion [69].

Moreover, it has been well known for a long time that the insulin concentration required to suppress lipolysis is far lower than what is needed to promote glucose utilization, although the hypoglycemic activity of insulin does not have the “threshold” phenomenon, while its antilipolytic and antiketotic activities do [70].

In the kidney, there are several other mechanisms that have been assumed to be implicated in the etiopathogenesis of euDKA. Indeed, SGLT2s are members of a wide family of active sodium/glucose co-transporters: SLC5 (formed by 12 different proteins) [71]. One of these SLC5 transporters is sodium-coupled monocarboxylate transporter-2 (SMCT-2), the reabsorbed ketones in the S1 segment of kidney proximal tubules. The SMCT-2, belonging to the same family of receptors, has some similarities with SGLT2’s structure, and it is conceivable that an interaction between these proteins and the SGLT2-i can occur [72]. In addition, the urate anion transporter 1 (URAT-1) is co-expressed with SMCT-2; these co-transporters reabsorb filtered urate in exchange with ketones or lactate, and their function might be altered by SGLT2-i action on SMCT-2 [73].

On the other hand, when SGLT-2 activity is inhibited by drugs, a reduction in the energy expended by the basolateral Na^+^/K^+^-ATPase occurs, promoting the sodium gradient across the apical membrane [74,75]. This process determines the increase in the concentration of sodium ions in the renal tubular lumen, with a consequent increase in the positive electrical charges that lead to a reduced urinary elimination of the negatively charged ketones [76,77]. Moreover, by reducing the renal glucose excretion threshold, SGLT2-i use may simulate a starvation status and induce an increase in ketone synthesis as a consequence of the metabolic shift from glucose to lipid use [78].

It must also be considered that it is not always easy to establish whether an SGLT2-i triggers directly euDKA or whether DKA occurs independently of these drugs, and an SGLT2-i simply reduces blood glucose dosage during the events (“cosmetic effect” on glycemia) [66]. 

Although there are several hypotheses to explain the occurrence of euDKA in subjects treated with an SGLT2-i, more studies are certainly necessary to further clarify the involved processes.

### Precipitating Factors

Even if the etiopathogenetic of euDKA induced by an SGLT2-i is not yet clear, an important role is played by a long list of precipitating factors, such as concurrent pathologies, infection, sepsis, dehydration, acute cardio-vascular events, a reduction in caloric and/or fluid intake, low-carbohydrate diet, a significant reduction in the daily insulin dose in insulin-treated patients and surgery. It is important to highlight that many of these conditions may independently predispose one to DKA, and the simultaneous administration of an SGLT2-i would further increase the risk of euDKA [79]. Therefore, clinicians must be able to recognize these possible trigger conditions and evaluate the suspension of SGLT2-i administration in the presence of precipitating factors, in order to reduce the possibility that euDKA can develop [15,80].

Overall, in the presence of signs/symptoms of ketonemia in patients on an SGLT2-i triggered by a possible precipitating factor [18], the euDKA diagnosis must be considered and sought by blood chemistry (the determination of ketones blood levels, blood gas analysis to assess the presence of metabolic acidosis, dosage of serum electrolytes, etc.). If the diagnosis is confirmed, it is necessary to suspend SGLT2-i administration and to ensure adequate therapy [15].

The most frequent etiopathogenetic mechanisms through which these conditions trigger the onset of euDKA are insulin resistance induction, the secretion of counterregulatory hormones (adrenaline, glucagon, etc.), catabolic state activation, increased peripheral glucose utilization and/or lowered glycogen stores and decreased carbohydrate levels intake that, in turn, lead to osmotic diuresis [81,82,83,84,85].

The main precipitating factors and their etiopathogenetic mechanisms are summarized in Table 4.

As already mentioned above, although the recent introduction of an SGLT2-i in the treatment of T2DM patients has focused attention on the correlation between euDKA and the use of these drugs, some of the above-listed conditions could cause this form of DKA regardless of the SGLT2-i administration. Among these, the most common cause is alcoholic ketoacidosis: in an alcoholic, a ketoacidosis without a raised glucose level is virtually diagnostic of alcoholic ketoacidosis. Alcohol-induced ketone production is characterized by a significant shift in production towards β-hydroxybutyric acid compared with acetoacetate (7:1 ratio). For this reason, if alcoholic ketoacidosis is suspected, capillary β-hydroxybutyrate should be measured and not urine ketone bodies as a suppression of acetoacetate production is suspected [85].

About surgery-related euDKA risk, a temporary interruption of SGLT2-i therapy is suggested by the European Medicines Agency at least 3 days after major surgical procedures in addition to the use of insulin for blood sugar management [86,87]. Insulin administration instead of SGLT2-i use in order to prevent DKA is also recommended for patients hospitalized due to a serious illness [86]. 

Also, pregnancy could be a risk factor for euDKA: indeed, the incidence of euDKA is significantly higher in pregnant than in non-pregnant women (8.9% vs. 3.1%) [92]. Regardless, the discussion of this topic is not among the objectives of this paper because, to date, SGLT2-i use is not indicated in pregnancy.

Initially, numerous reports demonstrated that a high number of DKA cases related to SGLT2-i administration involves patients with T1DM; in these patients, insulin deficiency was considered the major trigger for the development of euDKA. Subsequently, it was seen that T2DM patients may also develop this complication. Recently, emerging evidence seems to indicate that some of these cases were diagnosed in patients with an undiagnosed form of LADA [88]. 

At this regard, a review of the literature also suggests that an increased risk of ketoacidosis is present in T2DM patients with low C-peptide levels, particularly if therapy with statins and diuretics coexists, due to the further risk of hypokalemia and impaired release of insulin [76,89]. Several authors have highlighted how the risk for ketoacidosis with an SGLT2-i “*may be increased in long-standing T2DM patients with marked β-cell insufficiency or in latent autoimmune diabetes in adults with rapid evolution toward T1DM*” [65].

Currently, there are no recommendations in the guidelines about the benefits of antibody testing or screening after starting SGLT2-i administration to identify which patients may be at risk. Some authors recommend LADA screening for patients with at least two of the following criteria:

- Age of onset younger than 50 years;
- Acute symptoms (e.g., polyuria, polydipsia, unintentional weight loss);
- Body mass index less than 25 Kg/m^2^;
- A personal history of autoimmune disease;
- A family history of autoimmune disease.

Patients presenting two of these criteria should be further evaluated through testing the blood level of the C-peptide and GAD antibody titers [93,94]. 

However, recently, the ADA recognized that the current classification system presents challenges in the diagnosis and treatment of DM. The ADA has proposed that in the DM diagnosis, it should be important to focus attention towards β-cells’ normal functioning. This new approach is based on the concept that DM originates from abnormal pancreatic β-cells. This “β-cell-centric model” could provide a more correct diagnosis of LADA and easily indicate subjects at risk of developing euDKA under SGLT2-i treatment [90,91].

Once LADA is diagnosed, an SGLT2-i should be avoided or used with caution under closer monitoring to reduce the risk of DKA [93].

Furthermore, some authors suggested that 50% of diabetic ketoacidosis was present in subjects with anti-GAD65 autoantibodies, suspected to suffer from adult-onset autoimmune-mediated diabetes rather than non-autoimmune T2DM [43].

## 4. Management

euDKA is particularly insidious because it often has a latent onset, associated with unspecific symptomatology, until progressing to severe forms, complicated by systemic symptoms. A timely diagnosis is crucial to avoid its evolution into severe forms [18]. The first step is to stop SGLT2-i administration immediately; otherwise, euDKA is treated as hyperglycemic ketoacidosis by insulin infusion, the correction of electrolytes and dehydration [87]. 

The continuous intravenous infusion of insulin is started and an intensive subcutaneous regime is provided only when the acid–base equilibrium is restored and the anion gap corrected [95]. 

Insulin infusion should be started using a fixed rate of 0.1 units/kg/h. The targets are as follows:

- A reduction in the blood ketone concentration by 0.5 mmol/L/h;
- An increase in venous bicarbonate by 3.0 mmol/L/h;
- Lowering capillary blood glucose by 3.0 mmol/L/h;
- Maintaining potassium between 4.0 and 5.5 mmol/L.

In case of a failure, the fixed-rate intravenous insulin infusion rate should be increased.

Once the glucose blood level drops to <14 mmol/L, one should initiate dextrose (5% or 10%) straight away at 125 mL/h. 

If glycemia falls to <14 mmol/L despite glucose infusion, one should reduce the intravenous insulin infusion rate to 0.05 units/kg/h to avoid hypoglycemia [15].

Although euDKA management is considered to overlap with that of DKA, recent evidence suggests that in the case of euDKA, ketoacidosis is reversed with higher rates of glucose and insulin than those requested on the basis of blood glucose. Prospective trials are required to optimize the levels of glucose and insulin infusions in euDKA [96].

The restoration of circulatory volume and ketones clearance can be obtained with fluid replacement. All fluid loss (and the liquid requirement) should be replaced in 24–48 h [15]. Crystalloid rather than colloid solutions are recommended for fluid resuscitation [97]: the guidelines recommend a fluid 0.9% sodium chloride solution. However, when the glucose blood level falls below 14.0 mmol/L, a dextrose infusion is suggested to prevent hypoglycemia and to act as the substrate for insulin [98].

Despite this, according to some recent results from randomized trials, the use of balanced crystalloid solutions, compared to the commonly used 0.9% sodium chloride solution, may provide better outcomes for DKA patients, resulting more quickly in a lower resolution of acidosis, lower hyperchloremia and shorter hospitalization [99].

In patients with DKA, a potassium deficiency (usually 2–5 mmol/kg) is present, even if the serum potassium levels are high as the intracellular potassium passes into extracellular space, owing to insulin absence and high plasma osmolarity. Once insulin infusion has been initiated, potassium shifts back into the cells and its serum level drops. 

Potassium is osmotically active, so it must be infused carefully in patients with high serum osmolarity, (e.g., 40 mEq of potassium in one liter of an isotonic solution) [7]. 

According to the current recommendations, no potassium infusion must be implemented if the serum potassium level remains above 5.5 mmol/L in consideration of the possibility of acute pre-renal kidney injury related to severe dehydration. Potassium administration must be prescribed until the serum potassium level is below 5.5 mmol/L and urine is produced; if the serum potassium level falls below 3.5 mmol/L, the potassium regimen must be reconsidered. Instead, it is necessary to warrant that potassium levels are above 3.3 mEq/L before the administration of insulin [100,101,102].

Some papers have reported that bicarbonate, in patients with DKA, made no difference in the resolution of acidosis or time to discharge, and its use is generally not recommended. Its administration is only indicated for pH levels <6.9 to temporarily correct acidosis. When bicarbonate is infused, the administration of calcium intravenously is suggested to prevent hypocalcemia [97,103].

To date, no data suggest a positive role of routine phosphate replacement, unless there is a severe phosphate deficiency able to worsen respiratory failure and cause cardiac arrhythmias and rhabdomyolysis [15,104]. 

The euDKA resolution is defined as pH >7.3 units, bicarbonate >15.0 mmol/L and blood ketone level <0.6 mmol/L [8].

Implementing these supportive measures could safely restore a physiologic glucometabolic and acid–base equilibrium in most patients. SGLT2-i administration should be carefully evaluated after ascertaining the clinical picture and discovering the euDKA cause, according to the patient’s performance status and medical judgment [37].

## 5. Conclusions

Currently, the “real world” euDKA incidence in T2DM patients treated with an SGLT2-i cannot be determined in a certain way. From the data reported in clinical trials and observational studies, the incidence seems to be relatively low, but a real estimate is hampered by the absence of univocal diagnostic criteria and the heterogeneity of the study populations, which result in conflicting results. Furthermore, clinical trials and observational studies evaluating the association between SGLT2-i use and DKA risk have reported contradictory results. Even the etiopathogenetic mechanism of euDKA in patients taking an SGLT2-i is not fully understood yet, although an important role is surely played by precipitating factors, such as infections, concurrent pathologies, the post-operative stage, a reduction in caloric and/or fluid intake, alcohol abuse, a low-carbohydrate diet and a significant reduction in the daily insulin dose. As a result, clinical scenarios can be multifaceted and not easy to recognize. 

The use of these drugs is rapidly increasing worldwide, thanks to their effectiveness on glycemic control, numerous cardiovascular risk factors and their documented cardio-renal benefits. Therefore, it is essential that both physicians and patients are aware of the relatively small risk of euDKA associated with the use of SGLT2i as well as the main predisposing factors and its heterogeneous clinical presentation. Although rare, euDKA is a life-threatening complication. Therefore, it is important to spread knowledge of the risk of euDKA and its correct clinical approach, which involves the suspension of SGLT2i treatment, insulin infusion and an adequate intake of fluids in the presence of symptoms/signs of hyperketonemia, to avoid its evolution towards more severe forms. Thus, a timely diagnosis is essential.

Clinicians who treat diabetic patients with an SGLT2-i are also responsible for their education about the mechanisms and symptoms of euDKA. Furthermore, it is not always easy to discern whether an SGLT2-i triggers directly euDKA or if the latter is induced independently of these drugs, with the SGLT2-i only responsible for the reduction in the blood glucose levels during the events. For these reasons, upon suspecting euDKA induced by an SGLT2-i, a thorough differential diagnosis to rule out other possible misunderstood pathologies which could be the true etiology of euDKA is necessary, especially an undiagnosed LADA or other conditions characterized by reduced insulin secretion from pancreatic beta cells.

Although we are aware that the present review cannot be exhaustive, we believe that one of its strengths is the inclusion of data from the main and recent cardiovascular and renal outcome trials on SGLT2-i use. 

Furthermore, we have highlighted the urgent need for unique and universally recognized diagnostic criteria for euDKA.

In conclusion, adequate clinical and behavioral measures allow the prevention of euDKA; in our opinion, the awareness of the risk will not limit but facilitate a wider and safer use of this class of drugs, which has demonstrated to ensure glycemic and cardio-renal advantages, in large and heterogeneous populations.

However, more clinical information is requested on different clinical pictures and conditions in which euDKA cases in patient administered with an SGLT2-i may occur. Therefore, further knowledge on the metabolic and humoral effects of an SGLT2-i should help to obtain more solid basis for the safety and appropriate administration of this class of drugs. Undoubtedly, the challenge in recent research on combined, multifaceted approaches (plant-based diets, nutraceuticals and drugs) and their use as efficient strategies to prevent and treat DM and its comorbidities is always open [105].

## Figures and Tables

**Table 1:** DKA diagnostic criteria in adults. Comparison between JBDS guidelines, ADA guidelines and AACE/ACE guidelines and AMD, SID and SIEDP consensus statement.

		JBDS Guidelines	ADA Guidelines			AACE/ACE Guidelines	AMD, SID and SIEDP Consensus Statement		
			Mild	Moderate	Severe		Mild	Moderate	Severe
**“D”** **Glucose**		>11.0 mmol/L (200 mg/dL)	>13.9 mmol/L (>250 mg/dL)	>13.9 mmol/L (>250 mg/dL)	>13.9 mmol/L (>250 mg/dL)	^___^	>11.0 mmol/L (200 mg/dL)	>11.0 mmol/L (200 mg/dL)	>11.0 mmol/L (200 mg/dL)
**“K”** **Ketones**		>3.0 mmol/L (or history of diabetes)	Ketone-positive urine or serum	Ketone-positive urine or serum	Ketone-positive urine or serum	Ketone- positive serum ≥40 mg/dL (3.8 mmol/L) or urine	Ketonemia ≥3 mmol/L	Ketonemia ≥3 mmol/L	Ketonemia ≥3 mmol/L
							Ketonuria ≥2+	Ketonuria ≥2+	Ketonuria ≥2+
**“A”** **Acidosis**	**pH**	<7.3	7.25–7.3	7.0–7.24	<7.0	<7.3	<7.3	<7.2	<7.1
	**HCO_3_** **(mmol/L)**	<15	15 to 18	10 to 15	<10	^__^	<15	<10	<5
	**Anion gap** **(mEq/L)**	^___^	>10	>12	>12	>10	^___^	^___^	^___^
**Mental Status**		^___^	Alert	Alert or drowsy	Stupor or coma	Drowsy, stupor or coma	^___^	^___^	^___^

**Table 2:** Currently criteria for euDKA diagnosis.

	Munro’s Classic Definition	JBDS Guidelines
**“D” Glucose**	<16.6 mmol/L (300 mg/dL)	<13.8 mmol/L (250 mg/dL)
**“K” Ketones**	^___^	Ketonemia ≥31.6 mg/dL (3.0 mmol/L) Ketonuria (2+ or more on standard urine sticks)
**“A” Acidosis**	Raised anion gap metabolic acidosis	HCO_3_^−^ <10 mEq/l
**Medical History**	^___^	Known diabetes history

**Table 3:** Comparison of the incidence of DKA with SGLT2-i vs. placebo in major randomized controlled trials.

TRIAL	SGLT2-i	Incidence in SGLT2-i Groups	Incidence in Placebo Groups	Hazard Ratio (95% CI)	*p* Values
***EMPAREG-OUTCOME***	Empagliflozin 10 mg Empagliflozin 25 mg	1/2345 (<0.1%) 3/2342 (0.1%)	1/2333 (<0.1%)	^___^	NS
***EMPEROR-Preserved***	Empagliflozin 10 mg	4/1465 (0.3%)	5/1471 (0.3%)	^___^	^___^
***EMPA-KIDNEY***	Empagliflozin 10 mg	6/3304 (0.2%) 0.09/100 patients-yr	1/3305 (<0.1%) 0.02/100 patients-yr	^___^	^___^
***DECLARE-TIMI 58***	Dapagliflozin 10 mg	27/8574 (0.3%)	12/8569 (0.1%)	2.18 (1.10–4.30)	0.02
***DAPA-HF***	Dapagliflozin 10 mg	3/2373 * 0.1/100 patients-yr	0/2371	^___^	^___^
***DELIVER***	Dapagliflozin 10 mg	0/3132	2/3131 (<0.1%)	^___^	^___^
***DAPA-CKD***	Dapagliflozin 10 mg	0/2149	2/2149 (<0.1%)	^___^	0.5
***CANVAS***	Canagliflozin 100 mg Canagliflozin 300 mg	0.6/1000 patients-yr	0.3/1000 patients-yr	2–33 (0.76–7.17)	0.14
***CREDENCE***	Canagliflozin 100 mg	11/2200 2.2/1000 patients-yr	1/2197 0.2/1000 patients-yr	10.8 (1.39–83.65)	NA **
***VERTIS-CV***	Ertugliflozin 5 mg Ertugliflozin 15 mg	7/2746 (0.3) 12/2747 (0.4)	2/2745 (0.1)	^___^	^___^

NS: not statistically significant. NA: not available. * All cases of diabetic ketoacidosis occurred in patients with diabetes at baseline [30]. ** denotes not applicable because *p* values were reported only for outcomes that were included in the hierarchical testing strategy, and hazard ratios and 95% confidence intervals (CIs) are reported only for outcomes with more than 10 events [29].

**Table 4:** EuDKA precipitating factors and their etiopathogenetic mechanisms.

Precipitating Factors	Etiopathogenetic Mechanisms
Prolonged physical activity or exercise [70]	Increased counterregulatory hormones Increased peripheral glucose utilization Decreased carbohydrate intake
Decompensated cirrhosis [70,81]	Altered hepatic carbohydrate metabolism Decreased glycogen stores Catabolic state
Organic pancreatic insufficiency (acute or chronic pancreatitis) [70,81]	Reduced circulating insulin levels Fasting (in the case of acute pancreatitis)
Fasting [70,83]	Decreased carbohydrate intake causes insulinopenia and increased glucagon levels Decreased glycogen stores
Acute cardio-vascular events (ACS or stroke) [81]	Decreased carbohydrate intake Increased circulating levels of counterregulatory hormones
Cocaine use [81]	Decreased carbohydrate intake Increased circulating levels of counterregulatory hormones
Trauma [81]	Decreased carbohydrate intake Increased circulating levels of counterregulatory hormones Blood glucose dilution by large fluid shifts during resuscitation
Infection, sepsis [81,83,84]	Insulin resistance due to counterregulatory hormones (adrenaline, glucagon, etc.) Increased peripheral glucose utilization Catabolic state Decreased carbohydrate intake (nausea, vomiting, anorexia)
Excessive alcohol use [81,85]	Decreased carbohydrate intake Osmotic diuresis
Surgery [86,87]	Peri-operative fasting Gastrointestinal surgery has a higher incidence as fasting is prolonged and/or intestinal absorption is slow Increased counterregulatory hormones in post-operative stage
LADA [88,89,90,91]	Decreased/absent insulin secretion Decreased glycogen stores Osmotic diuresis Increased FFA metabolism

## Data Availability

No new data were created or analyzed in this study.
